# Lack of additive role of ageing in nigrostriatal neurodegeneration triggered by α-synuclein overexpression

**DOI:** 10.1186/s40478-015-0222-2

**Published:** 2015-07-25

**Authors:** Mathieu Bourdenx, Sandra Dovero, Michel Engeln, Simone Bido, Matthieu F. Bastide, Nathalie Dutheil, Isabel Vollenweider, Laetitia Baud, Camille Piron, Virginie Grouthier, Thomas Boraud, Grégory Porras, Qin Li, Veerle Baekelandt, Dieter Scheller, Anne Michel, Pierre-Olivier Fernagut, François Georges, Grégoire Courtine, Erwan Bezard, Benjamin Dehay

**Affiliations:** University de Bordeaux, Institut des Maladies Neurodégénératives, UMR 5293, 33000 Bordeaux, France; CNRS, Institut des Maladies Neurodégénératives, UMR 5293, 33000 Bordeaux, France; International Paraplegic Foundation chair in spinal cord Repair, Center for Neuroprosthetics and Brain Mind Institute, School of Life Sciences, Swiss Federal Institute of Technology (EPFL), Lausanne, Switzerland; Motac Neuroscience, Manchester, UK; Institute of Laboratory Animal Sciences, China Academy of Medical Sciences, Beijing, China; Department of Neurosciences and Leuven Research Institute for Neuroscience and Disease (LIND), KU Leuven, Laboratory for Neurobiology and Gene Therapy, Kapucijnenvoer 33, box 7001, 3000 Leuven, Belgium; Parkinson’s Disease Pharmacology, UCB Pharma S.A., Braine L’Alleud, B-1420 Belgium; University de Bordeaux, Interdisciplinary Institute for Neuroscience, UMR5297, Bordeaux, France; CNRS, Institut Interdisciplinaire de Neurosciences, UMR 5297, F-33000 Bordeaux, France

**Keywords:** Ageing, α-synuclein, Animal models, Adeno-associated viral vector, Parkinson’s disease

## Abstract

**Introduction:**

Parkinson’s disease (PD) is a progressive neurodegenerative disorder characterized by the loss of dopaminergic neurons as well as the presence of proteinaceous inclusions named Lewy bodies. α-synuclein (α-syn) is a major constituent of Lewy bodies, and the first disease-causing protein characterized in PD. Several α-syn-based animal models of PD have been developed to investigate the pathophysiology of PD, but none of them recapitulate the full picture of the disease. Ageing is the most compelling and major risk factor for developing PD but its impact on α-syn toxicity remains however unexplored. In this study, we developed and exploited a recombinant adeno-associated viral (AAV) vector of serotype 9 overexpressing mutated α-syn to elucidate the influence of ageing on the dynamics of PD-related neurodegeneration associated with α-syn pathology in different mammalian species.

**Results:**

Identical AAV pseudotype 2/9 vectors carrying the DNA for human mutant p.A53T α-syn were injected into the substantia nigra to induce neurodegeneration and synucleinopathy in mice, rats and monkeys. Rats were used first to validate the ability of this serotype to replicate α-syn pathology and second to investigate the relationship between the kinetics of α-syn-induced nigrostriatal degeneration and the progressive onset of motor dysfunctions, strikingly reminiscent of the impairments observed in PD patients. In mice, AAV2/9-hα-syn injection into the substantia nigra was associated with accumulation of α-syn and phosphorylated hα-syn, regardless of mouse strain. However, phenotypic mutants with either accelerated senescence or resistance to senescence did not display differential susceptibility to hα-syn overexpression. Of note, p-α-syn levels correlated with nigrostriatal degeneration in mice. In monkeys, hα-syn-induced degeneration of the nigrostriatal pathway was not affected by the age of the animals. Unlike mice, monkeys did not exhibit correlations between levels of phosphorylated α-syn and neurodegeneration.

**Conclusions:**

In conclusion, AAV2/9-mediated hα-syn induces robust nigrostriatal neurodegeneration in mice, rats and monkeys, allowing translational comparisons among species. Ageing, however, neither exacerbated nigrostriatal neurodegeneration nor α-syn pathology per se. Our unprecedented multi-species investigation thus favours the multiple-hit hypothesis for PD wherein ageing would merely be an aggravating, additive, factor superimposed upon an independent disease process.

**Electronic supplementary material:**

The online version of this article (doi:10.1186/s40478-015-0222-2) contains supplementary material, which is available to authorized users.

## Introduction

Parkinson’s disease (PD) is characterized by a profound loss of dopaminergic neurons from the substantia nigra, pars compacta (SNpc) associated with the presence of intraneuronal proteinaceous inclusions, named Lewy bodies (LB) in cell bodies and Lewy neurites (LN) in neuritis [1]. Although several genetic loci have been linked to the development of PD, ageing remains the most prominent risk factor [2]. Two main hypotheses have been formulated regarding the influence of ageing: one proposes that ageing increases dopamine neuron susceptibility [3] and the other that PD arises from the combination of several insults over the individual’s life [4]. Overall, the impact of ageing has been largely overlooked in preclinical studies of PD.

A major protein component of LB and LN is α-synuclein (α-syn), a synaptic protein of, as yet, unclear function [5]. Interestingly, α-syn has a unique importance in the aetiology of PD, as it appears to link familial and sporadic forms of the disease [6]. Several α-syn transgenic mice have been developed, expressing human wild type (WT) or mutated α-syn under different promoters [7]. They failed, however, to reproduce the combination of progressive and specific dopamine cell loss, protein aggregation and manifestation of parkinsonian-like symptoms [8]. Subsequently, the development of lentiviral and recombinant adeno-associated viral vectors (AAV) has opened up new possibilities regarding disease modelling. Nevertheless, the literature reports a bewildering variety of vectors, expression cassettes, serotypes, and titers, that hampers comparison between strains, species and investigators [8–10].

We, therefore investigated the spatial and temporal dynamics of dopamine neuron degeneration, human mutant p.A53T α-syn (hα-syn) expression and the resulting synucleinopathy using AAV pseudotype 2/9 (AAV2/9) in different species, namely mouse, rat and marmoset monkey. After replicating the progressive degeneration in rats and characterizing their parkinsonian-like phenotype, we questioned whether ageing influences susceptibility of dopamine neurons to neurodegeneration using senescence-prone and -resistant mouse strains and a population of marmoset monkeys featuring young and aged individuals. Our results demonstrate that ageing does not amplify the neurodegenerative process but simply adds to the α-syn overexpression supporting the multi-hit hypothesis.

## Materials and methods

### Adeno-Associated Viral vector production

Recombinant AAV2/9-p.A53T-human-α-syn (AAV-hα-syn) vectors were produced by polyethylenimine (PEI) mediated triple transfection of low passage HEK-293 T /17 cells (ATCC; cat number CRL-11268). The AAV expression plasmid pAAV2-CMVie/hSyn-synA53T-WPRE-pA (provided by Dr V. Baekelandt) was co-transfected with the adeno helper pAd Delta F6 plasmid (Penn Vector Core, cat # PL-F-PVADF6) and AAV Rep Cap pAAV2/9 plasmid (Penn Vector Core, cat # PL-T-PV008).

AAV vectors were purified as previously described [11]. Cells are harvested 72 h post transfection, resuspended in lysis buffer (150 mM NaCl, 50 mM Tris–HCl pH 8.5) and lysed by 3 freeze-thaw cycles (37 °C/-80 °C). The cell lysate is treated with 150units/ml Benzonase (Sigma, St Louis, MO) for 1 h at 37 °C and the crude lysate is clarified by centrifugation. Vectors are purified by iodixanol step gradient centrifugation, and concentrated and buffer-exchanged into Lactated Ringer’s solution (Baxter, Deerfield, IL) using vivaspin20 100 kDa cut off concentrator (Sartorius Stedim, Goettingen, Germany).

Titrations were performed at the transcriptome core facility (Neurocentre Magendie, INSERM U862, Bordeaux, France). The genome-containing particle (gcp) titer was determined by quantitative real-time PCR using the Light Cycler 480 SYBR green master mix (Roche, cat # 04887352001) with primers specific for the AAV2 ITRs (fwd 5’-GGAACCCCTAGTGATGGAGTT-3’; rev 5’-CGGCCTCAGTGAGCGA-3’) [12] on a Light Cycler 480 instrument.

Purity assessment of vector stocks was estimated by loading 10 μl of vector stock on 10 % SDS acrylamide gels, total proteins were visualized using the Krypton Infrared Protein Stain according to the manufacturer’s instructions (Life Technologies).

### Animals

Experiments were performed in accordance with the European Union directive of September 22, 2010 (2010/63/EU) on the protection of animals used for scientific purposes. The Institutional Animal Care and Use Committee of Bordeaux (CE50) approved experiments under the license number 5012099-A (rodents) and 50120102-A (monkeys). Kinematics experiments were performed under the guidelines established at EPFL. Local Swiss Veterinary Offices approved all the procedures. Experiments were performed on Sprague Dawley (53 animals), Wistar (8 animals) and Lewis rats (10 animals) with initial weight of approximately 200 g and age of 8 weeks. Rats were ordered from Janvier Labs or Charles River in France. Mouse experiments were performed on C57Bl/6 J (12 animals – ordered from Charles River laboratories), SAMR1 (19 animals) and SAMP8 (21 animals). Both SAMR1 and SAMP8 were purchased from the research animal facility of the Barcelona Science Park (Barcelona, Spain).

Monkey experiments were performed in an Association for Assessment and Accreditation of Laboratory Animal Care accredited facility following acceptance of study design by the Institute of Lab Animal Science (Chinese Academy of Science, Beijing, China) Institutional Animal Care and Use Committee. Thirteen male marmoset monkeys (*Callithrix jacchus*; Beijing, People’s Republic of China) were housed in 2 primate cages, allowing visual contacts and interactions with monkeys housed inside the cage. The use of wood chip or shredded paper litter on the cage floor is a source of environmental enrichment. Food and water were available ad libitum and animal care was supervised daily by veterinarians skilled in the health care and maintenance of nonhuman primates.

### Rodent experiments

#### 1-Methyl-4-phenyl-1,2,3,6-tetrahydropyridine-intoxicated mice

Eight- to 10-week-old male C57BL/6 J, SAMP8 and SAMR1 mice received one intraperitoneal injection of 1-methyl-4-phenyl-1,2,3,6-tetrahydropyridine (MPTP)-HCl per day (30 mg/kg free base; Sigma) for five consecutive days. Control mice received saline injections only. Mice were killed five months after MPTP intoxication. Four to nine mice were used in each group.

#### Surgical procedures

All the interventions were performed under full general anesthesia with isoflurane in oxygen-enriched air (1-2 %). After surgery, rodents were placed in an incubator for optimized recovery from anesthesia. Rats were injected either bilaterally (kinematic recordings,) or unilaterally (immunohistochemical investigation and basic motor behavior (spontaneous locomotor activity and stepping test)) in the SNpc with the AAV-hα-syn (2 μl - 7.0 ×10^12^ vg/ml) or the control AAV-GFP (7.0 ×10^12^ vg/ml – solely for kinematic recordings). Under isoflurane anesthesia, rats were placed in a stereotaxic frame (Kopf Instruments) and received two bilateral intranigral injections (Anteroposterior: −4.9 and −5.4; Mediolateral: ± 2.2 and ± 2; Dorsoventral: −7.8, in mm from bregma) of either vector, as previously described [13]. Mice were injected unilaterally with the AAV-hα-syn (120 nl - 7.0 ×10^12^ vg/ml) in the right SNpc (coordinates from Bregma: AP = −2.9, L = −1.3, DV = −4.5). Viruses were injected with a glass pipette at 0.4 μl.min^−1^ and the pipette was left in place for 4 min after injection to avoid leakage.

#### Kinematic Recordings

All procedures used have been detailed previously [14]. Briefly, whole-body kinematics were recorded using the high-speed motion capture system Vicon (Vicon Motion Systems, UK), combining 12 infrared cameras (200 Hz). 4 mm reflective markers were attached bilaterally overlying the iliac crest, the greater trochanter (hip), the lateral condyle (knee), the malleolus (ankle), and the base of the metatarsal phalageal joint (MTP). 3D position of the markers was reconstructed offline using Vicon Nexus software (1.8). The body was modeled as an interconnected chain of rigid segments, and joint angles were generated accordingly. For subsequent kinematic analysis, only hindlimb and parameters related to the trunk were analyzed. Parameters (Additional file 1: Table S1) describing gait timing, joint kinematics, limb endpoint trajectory, and trunk stability were computed for each gait cycle using custom written MATLAB scripts and according to methods described previously [14].

#### Behavioral Tasks

##### Spontaneous locomotor actimetry

Spontaneous locomotor activity was evaluated in cages similar to home cages (35x25x25 cm) flanked with photobeam to allow computerized counting of horizontal beam breaks (protocol adapted from [15]. After a 3 h habituation session, each subsequent recording sessions (i.e., Bsl, 4, 8, 12, 16 weeks) lasted 3 h.

##### Stepping test

Forelimb akinesia was assessed using stepping [13, 16]. Briefly, animals were gently held and conducted over a 90 cm distance to allow forehand followed by backhand steps count. Left and right limb performances were successively evaluated over 2 daily sessions on 3 consecutive days. The average number of left/right forehand steps was averaged over the 6 sessions for each time-point.

##### Kinematic recordings

Rats were handled and trained during one week prior to surgery in order to accustom them to the two locomotor tasks. After AAV-hα-syn injection surgery animals were trained 2–3 times per week and monitored visually to assess whether PD-like symptoms had developed. Out of 8 rats initially injected 6 displayed observable bradykinesia and were thus included in the functional analysis. Symptoms emerged at different timepoints for the individual rats (8–16 weeks post injection) and the recordings used for analysis are from these chronic timepoints. Due to improved functional performance with training animals were only trained once a week after symptoms had emerged. Overground and ladder locomotion were recorded on different days. One rat was not included in the ladder analysis since not enough functional steps could be recorded from this animal in this task. AAV-hα-syn rats were compared to a set of healthy rats that were extensively trained to perform both locomotor tasks.

Overground locomotion was tested on a 15 cm wide and 120 cm long, elevated runway. 10–15 steps per side were analyzed per rat. A total of 89 kinematic parameters were computed and included in the subsequent PC analysis for this task (Additional file 1: Table S1).

Crossing of an elevated horizontal ladder (rung spacing: 5.2 +/− 0.3 cm) was tested in alternation with overground walking. 10–15 steps per side were analyzed per rat. Quantification of hit, slip or miss paw placement was assessed from slow motion videos acquired at 100 Hz.

#### Principal Component Analysis

Behavioral data was analyzed by PC analysis [17]. Not all the steps are depicted in the figures.

Step 1: Continuous locomotion is recorded using a high-resolution kinematic system.
Step 2: Custom-written MATLAB scripts are applied to reconstructed kinematic data in order to compute basic parameters and highly elaborated variables that provide a comprehensive quantification of gait features. All variables computed are specified in Additional file 1: Table S1. Approximately 10–15 steps were extracted per rat and experimental condition.
Step 3: The matrix combining all values of variables from all rats and steps was then subjected to a PC analysis. For this purpose, we used the correlation method, which adjusts the mean of the data to 0 and the SD to 1. This method allows the comparison of variables with disparate values (large versus small), and/or different variances. The result of the PC analysis is a new set of synthetic uncorrelated variables, i.e., the PCs, which each explains the maximum possible amount of variance.
Step 4: The new coordinates of gait patterns along each PC, termed PC scores, are extracted for each rat. PC scores are used to represent gait patterns in the “denoised” PC space to visualize differences between mice and experimental conditions.
Step 5: PC scores are averaged for each experimental condition and represented in histogram plots to identify the type of information differentiated along each PC axis.
Step 6: Each PC is a linear combination of the original parameters with appropriate weights, which are termed “factor loadings.” The values of factor loadings range from −1 to 1, and correspond to correlations between original parameters and a given PC.
Step 7: Factor loadings with a high value (|factor loading| > 0.5) are extracted, color-coded based on their correlation value, and regrouped into functional clusters based on the type of gait control aspects they refer to. This process leads to an objective extraction of the most relevant behavioral parameters to account for the effects of a specific experimental condition (Additional file 2: Figure S6).
Step 8: To provide a more classical representation of the observed effects, relevant parameters representative of functional clusters are extracted and represented in histogram plots.

### Primate experiments

#### Surgery

Eight two-years-old (young group) and five six-years-old (old group) common marmosets (*Callithrix jacchus*) were unilaterally injected with 4 μL AAV-hα-syn in 2 points of the SNpc following electrophysiological recordings (AP: +4 and +3.3 mm, L: +3.7 and +3.5 mm, D: +15 mm from interaural line). Recording system: 16 channel wireless system, Multichannel Systems, Reutlingen, BW, Germany). Animals were anaesthetized with atropine SO_4_ at 0.04 mg/kg, i.m. prior to preparation for surgery. At least 10 min later, the animals were anaesthetized with ketamine HCl at 10 mg/kg, IM. Following stereotaxic injections, viral expression was allowed for the next eleven weeks.

#### Post-mortem processing

At the end of different experiments, animals were killed by sodium pentobarbital overdose (150 mg/kg, i.v.), and perfused transcardiacally with 0.9 % saline solution (containing 1 % heparin) followed by 4 % PFA performed in accordance with accepted European Veterinary Medical Association guidelines. Brains were removed quickly after death and post-fixed overnight in the same fixative, then cryoprotected in PBS containing 20 % sucrose and frozen by immersion in a cold isopentane bath (−45 °C), before being stored at −80 °C until sectioning. Brains were sectioned in a Leica CM3050S cryostat (Leica Microsystems, Wetzlar, Germany) at −20 °C. Brains were cut at 50 μm-thick sections and both striatal and SNpc levels were collected.

#### Immunohistochemistry

##### Extent of lesion

To assess the integrity of the nigrostriatal pathway, tyrosine hydroxylase (TH) immunohistochemistry was performed on striatal and SN free-floating sections (Additional file 3: Table S2). On the rat experiment, dopamine transporter (DAT) immunochemistry on striatal sections was also done. Briefly, striatal sections from three representative levels (anterior, medial and posterior) were incubated with a mouse monoclonal antibody raised against human TH (Millipore, MAB318, 1/5 000) or rabbit polyclonal antibody raised against DAT ([18], 1/5 000) for 72 h at room temperature. The staining was revealed by a specific peroxidase EnVision™ system (mouse or rabbit HRP EnVison™ kit DAB+ DAKO, K4007 or K4011) followed by DAB visualization. Midbrain sections containing the SNpc were processed for tyrosine hydroxylase. Serial Free-floating sections were incubated in mouse monoclonal TH antibody (Millipore, MAB318, 1/5 000) for one night at room temperature and revealed by an anti-mouse peroxidase EnVision™ system followed by DAB staining. SN free floating sections are mounted on gelatinized slides, counterstain with 0.1 % cresyl violet solution, dehydrated and coversliped while striatal sections are mounted on gelatinized slides and coversliped only.

##### Human α-syn expression

Human α-syn expression levels were revealed in the striatum (anterior, median and posterior part) and in the SNpc by immunohistochemistry (Additional file 3: Table S2). The selected sections of the striatum, and serial free-floating sections of the SN were incubated with a mouse monoclonal antibody raised against human α-syn (clone syn211 Thermo Scientific, MS1572, 1:1000) for one night at room temperature and revealed by an anti-mouse peroxidase EnVision™ system (DAKO, K4007) followed by DAB incubation. Phosphorylated synuclein immunochemistry was investigated in the substantia nigra in mouse and marmoset experiments. Briefly, serial sections of the SN were incubated for 3 nights at room temperature in a rabbit anti p-Syn (Abcam AB51253 – 1/500) and revealed with the rabbit HRP EnVision polymere system™ followed by DAB as substrate. Free-floating sections were mounted on gelatinized slides, dehydrated and coverslipped before further analysis.

##### Fluorescence co-localization

Co-localization of Ubiquitin and TH with α-syn was revealed by a triple fluorescence immunohistochemistry within the SNpc of sham or AAV-hα-syn injected rat at 16 week post-surgery. All sections were individually identified to perform staining with all sections in the same conditions. After a blocking step in 5 % fat milk, TH was first revealed with a sheep anti TH (Abcam −1/1000) for one night followed by a donkey anti-sheep Alexa 488 (1/500-1 h). The staining was briefly observed under microscope before to continue with a simultaneous incubation of a mouse anti-α-syn and rabbit anti-Ubiquitin both diluted at 1/1000 in PBS. Then, the α-syn signal is revealed first by incubate sections in a Alexa Cys5 goat anti mouse for 1 h at RT and the double staining TH- α-syn was observed before the last staining with an anti-rabbit HRP EnVision™ system (DAKO, K4011) followed by an 549 Dylight anti HRP diluted at 1/1000 for 1 h. Free-floating sections were mounted on slides and coverslipped with fluoromount™ aqueous mounting media (Sigma, F4680).

#### Image analysis

##### Grey level TH quantification in the striatum

TH level in the striatum is quantified by densitometry. Sections are scanned in an Epson expression 10000XL high-resolution scanner and images are used in ImageJ software to compare the grey level in the delineated striatum for each animal.

##### Stereological assessment of TH positive cells in the SN

TH-positive SN cells were counted by stereology blind with regard to the experimental condition using a Leica DM6000B motorized microscope coupled with the Mercator Pro software (ExploraNova, La Rochelle, France).The SN was delineated for each section and probes for stereological counting were applied to the map obtained (sampling and size of probes, see Additional file 4: Table S3). Each TH-positive cell with its nucleus included in the probe or intersecting any of the acceptance lines was counted. The optical fractionator method was finally used to estimate the total number of TH-positive cells in the SN of each animal.

##### α-syn and DAT surface quantification in the STR

α-syn expression and DAT staining were quantified in the striatum on high-resolution images from NanoZoomer 2.0 HT (BIC facility). Each NDPI image obtained was used in Mercator Pro software (Explora Nova, La Rochelle, France) to quantify the representative surface of α-syn or DAT staining within the striatum. Briefly, striata were delineated and a color threshold was applied for each image using the same parameters allowing comparison between experimental groups. The surface detected was then compared to the whole striatum surface in order to obtain a percentage of stained structure.

##### α-syn and p-α-syn volume quantification in the SN

In order to quantify the expression of α-syn or p-α-syn in the whole SN, a semi-stereological assessment was used for each animal. As for the striatal α-syn quantification, all slides with serial sections of the whole SNpc were scanned in a NanoZoomer 2.0 HT (BIC facility). First, NDPI images obtained were analyzed in Mercator Pro software (Explora Nova, La Rochelle, France) to quantify the representative surface of the staining in each SN section using a color threshold. Then, surfaces detected were reported to the sampling scheme (Additional file 4: Table S3) to assess the representative volume of α-syn or p-α-syn expression for each SN using the Cavalieri method. Finally the percentage of SN volume stained for α-syn was used to compare the effect of each treatment.

## Results

### α-syn overexpression induces a progressive degeneration of the nigrostriatal pathway in rat

**Fig. 1:**
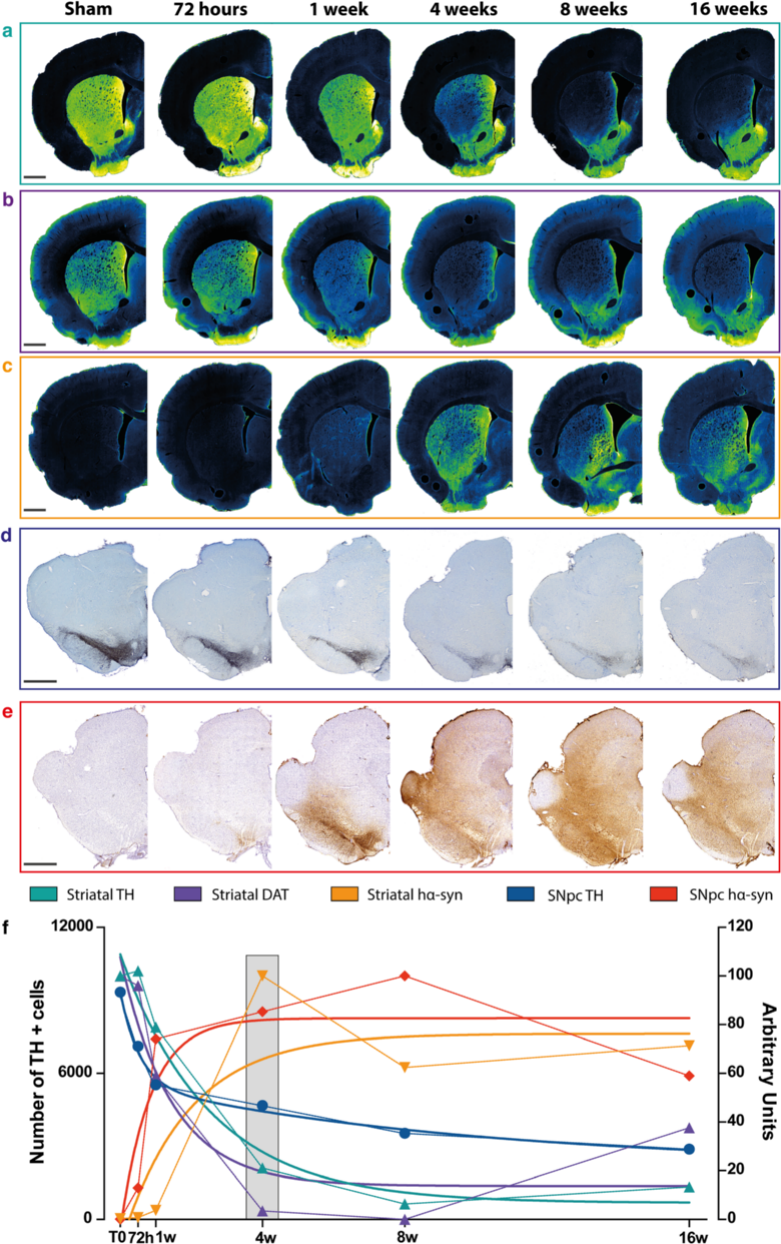
rAAV2/9 vector-mediated overexpression of hα-syn in rat SNpc induces progressive dopaminergic neurodegeneration related to hα-syn expression dynamics. (**a**-**e**) Representative photomicrographs of dopaminergic markers and human α-syn immunostaining at striatal (**a**-**c**) and SNpc (**d**,**e**) levels at several time points after injection. (**a**,**d**): tyrosine hydroxylase (TH); (**b**): dopamine transporter (DAT); (**c**,**e**): human α-syn. (f) Quantification of the different markers over time. Briefly, stereological quantification of the number of TH-positive cells in the SNpc of rat are reported on the left axis, all staining intensity have been normalized between min (0 %) and max (100 %) and reported on the right axis to allow comparison of the evolution. Dot-lines represent actual values while plain curves are regression curves. Colors are the same as in the upper part of the panel: light blue: striatal TH, purple: striatal DAT, yellow: striatal α-syn, blue: SNpc TH, red: SNpc α-syn. Scales applies to all pictures: 1 mm

To determine the course of dopamine neuron degeneration, adult Sprague–Dawley rats received intranigral stereotactic injection of AAV2/9 carrying human p.A53T α-syn (AAV-hα-syn). We then assessed several markers of the nigrostriatal pathway: tyrosine hydroxylase (TH) and dopamine transporter (DAT) immunoreactivities in the striatum as well as the number of TH-positive cells in the SNpc which all correlated with the protein expression of hα-syn in both striatum and SNpc at five time points (72 h, 1, 4, 8 and 16 weeks) (Fig. 1). Intranigral injection of AAV-hα-syn resulted in a markedly significant reduction of dopaminergic fibers, evidenced by a reduction of TH (Fig. 1a and f, Additional file 5: Figure S1a) and DAT (Fig. 1b and f, Additional file 5: Figure S1b) immunostaining, beginning at 1 week and reaching a plateau at 4 weeks, associated with progressive dopamine neuron death ranging from 50 % at 1 week to approximately 80 % at 16 weeks after surgery (Fig. 1d and f, Additional file 5: Figure S1c). A two-phase exponential decay equation models the time-course of SNpc dopaminergic neuron loss (*y* =2230 + 3707.2728e^-2.171x^ + 3417.877e^-0.1069x^, *r*^2^ = 0.66, Fig. 1f). These results indicate that nigral expression of hα-syn induces progressive dopamine neuron degeneration in rat, in agreement with previous findings [10, 19, 20].

We next attempted to examine the hα-syn expression pattern in SNpc and striatum over time. Coinciding with the onset of neurodegeneration (72 h after injection), we observed a progressive increase in hα-syn around the injection site at 72 h that peaked at 8 weeks post-injection (Fig. 1e and f, Additional file 5: Figure S1d). In the striatum, hα-syn staining was detected weakly at 1 week and reached its highest level at 4 weeks (Fig. 1c and f, Additional file 5: Figure S1e) concomitantly with the significant decrease in striatal dopamine markers (Additional file 5: Figure S1a and b).

Anatomically, expression of hα-syn started in the SNpc before being noticeable in the striatum (Additional file 6: Figure S2a and b). A strong intraneuronal hα-syn staining was observed at 72 h that then changed to a diffuse pattern in later time points (Additional file 6: Figure S2a). The striatum, on the other hand, showed long, wire-like, staining from 1 week onwards, subsequently changing to a more punctate form (Additional file 6: Figure S2b). In the SNpc, hα-syn co-localized with ubiquitin in neuronal cells at 8 weeks after injection (Additional file 7: Figure S3e-l), in contrast to sham-operated rats (Additional file 7: Figure S3a-d). Staining of hα-syn phosphorylated at S129 (p-α-syn), a common marker for pathological α-syn in post-mortem tissue [21], was observed specifically within SNpc neurons in AAV-hα-syn-injected rats at 16 weeks post-injection (Additional file 8: Figure S4b-e) but not in sham-operated rats (Additional file 8: Figure S4a). P-α-syn staining was mostly observed in perikarya of neuronal cells as well as in dendritic terminals (Additional file 8: Figure S4c-d). While hα-syn staining spread throughout the whole mesencephalon at 16 weeks after surgery (Fig. 1e), p-α-syn immunolabelling remained restricted to SNpc neurons (Additional file 8: Figure S4). These results indicate the presence of intracytoplasmic accumulation of α-syn, positive either for ubiquitin or for p-α-syn, in AAV-hα-syn-injected rats.

Because earlier work has shown differential rodent strain neurodegeneration susceptibility to neurotoxin [22], we then investigated whether two common laboratory rat strains, i.e., Sprague–Dawley and Wistar, would display a different susceptibility to hα-syn overexpression. Strikingly, Wistar rats were significantly less susceptible to dopamine neuron degeneration (45 % at 8 weeks) compared to Sprague–Dawley rats (69 % at 8 weeks) (Additional file 9: Figure S5), highlighting the importance of the genetic background in the response to hα-syn overexpression.

### α-syn overexpression induces a progressive motor dysfunction in rats

**Fig. 2:**
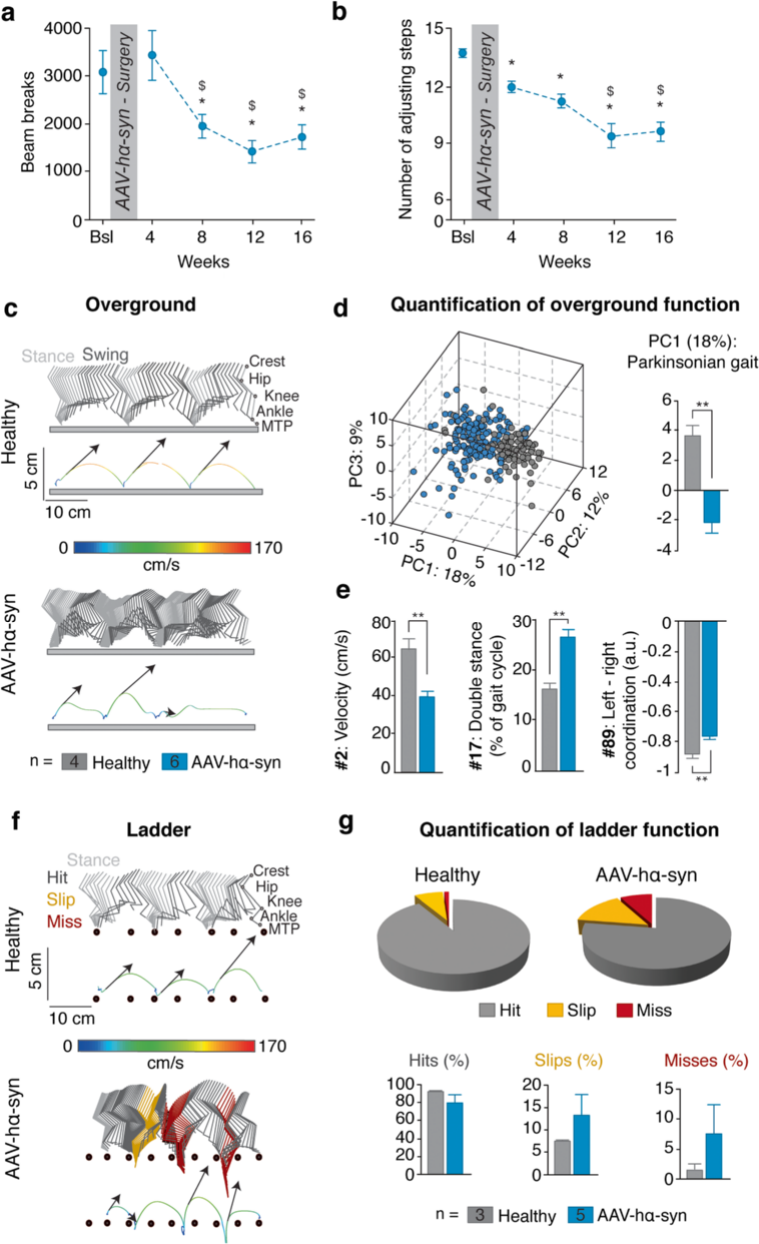
Behavioral impairments associated with hα-syn overexpression in rats. **a** Spontaneous locomotor activity at baseline (Bsl) and at 4, 8, 12 and 16 weeks after surgery. **b** Stepping test at baseline and at 4,8,12 and 16 weeks after surgery. **c** Color-coded stick diagram decomposition of hindlimb movement for a representative healthy and AAV-hα-syn rat during crossing of an elevated horizontal ladder with irregularly spaced rungs (spacing 5.2 +/− 0.3 cm). The corresponding endpoint trajectory is shown below. The colour code indicated the instantaneous velocity of the endpoint, while the arrows report the orientation and intensity of the foot push-off at swing onset. Light grey: stance; dark grey: hit; yellow: slip; dark red: miss. **d** Pie charts summarize total percentage of hits, slips, and misses (total of 182 steps evaluated, n are indicated in the legend boxes). Below percentage of hits, slips and misses are quantified as histogram plots. **e** The same representation is shown for a representative healthy and Parkinsonian rat during overground locomotion. **f** Principal component (PC) analysis was applied on 89 gait parameters measured during overground locomotion (10–15 gait cycles/hindlimb/rat, *n* = 4 healthy and *n* = 6 AAV-hα-syn rats). Each gait cycle is represented as a dots in the new space created by PC1–3. Histogram plot depicts mean values of PC1 scores for each experimental group. **g** Histogram plots represent mean values of parameters with high factor loading (|factor loading| > 0.5) on PC1, illustrating the most salient differences in motor control between the two groups. Numbers refer to Additional file 1: Table S1. *p < 0.05; **p < 0.01; ***p < 0.001; $ *p* < 0.05 vs 4 weeks time point; error bars, SEM; PC, Principal Component; a.u., arbitrary unit

The extent of lesion observed suggested that AAV-hα-syn-injected rats might develop overt parkinsonism. While AAV-hα-syn-injected rats displayed a significant decrease in spontaneous activity (Fig. 2a) from 8 weeks post-surgery onwards, they showed a significant decrease in the number of adjusting steps as early as 4 weeks and thereafter (Fig. 2b).

We then conducted high-resolution kinematic analyses to obtain quantified and detailed analyses of motor impairments. We first evaluated the rats during self-initiated overground locomotion. While sham rats fluidly crossed the runway at a fast pace, AAV-hα-syn-injected rats walked slower and exhibited a more variable and shuffling gait pattern (Fig. 2c). To measure these deficits, we computed 89 parameters providing quantitative measurements of bilateral gait features (Additional file 1: Table S1), and submitted all the parameters to a principal component (PC) analysis (*n* = 4 sham; *n* = 6 AAV-hα-syn rats; 10–15 steps/rat/side). This method allows the objective identification of the specific features that are affected or not affected by injuries or experimental conditions [23]. Representation of all data points in the de-noised space created by PC1-3 (Fig. 2d) showed that the gait patterns of healthy and AAV-hα-syn-injected rats clustered in distinct spaces that minimally overlapped. Analysis of individual scores on PC1, which explained 18 % of the total variance, revealed a significant difference between healthy and AAV-hα-syn-injected rats (*p* < 0.01; Fig. 2d). Thus, PC1 distinguished healthy from AAV-hα-syn-injected rats. We next extracted the parameters correlated with the AAV-hα-syn-specific PC1 (|factor loading| > 0.5), and regrouped them in functional clusters (Additional file 2: Figure S6). 38 % of all parameters analysed were altered in AAV-hα-syn-injected rats compared to healthy animals. Analyses of these parameters revealed that AAV-hα-syn-injected rats displayed altered gait timing, reduced control over limb endpoint trajectory and velocity, increase medio-lateral instability and aberrant inter-limb coordination (Fig. 2e).

We then tested rats during walking on a horizontal ladder, requiring precision in foot placement. Whereas sham rats progressed across the ladder with ease, AAV-hα-syn-injected rats crossed the ladder at a reduced velocity, with occasional periods of freezing (Additional file 10: Movie S1). They exhibited inaccuracy in the positioning of the hind paws onto the rungs compared to healthy rats (Fig. 2f). This misplacement resulted in an increased percentage of slips (healthy: 7.4 +/− 0.3 %; AAV-hα-syn rats: 13.1 +/− 4.7 %) and misses (healthy: 1.2 +/− 1 %; AAV-hα-syn: 7.6 +/− 4.8 %) in AAV-hα-syn-injected rats (Fig. 2g). While individual rats displayed variable behaviours on the ladder (Fig. 2g), this precision task uncovered more pronounced motor deficits compared to basic runway locomotion.

 Taken together, these data indicate that the hα-syn-induced nigrostriatal degeneration in the rat is accompanied by a progressive onset of motor dysfunctions reminiscent of the impairments observed in PD patients.

### Premature ageing mice did not present higher susceptibility to α-syn overexpression

Because ageing remains the most compelling risk factor for PD [2], we next investigated the relation between ageing and hα-syn-induced neurodegeneration using a panel of mice strains. The C57Bl/6 J, the senescence-accelerated mouse-prone 8 (SAMP8), the senescence-accelerated mouse-resistant 1 (SAMR1), and their littermate controls were used. The SAMP8 mice are naturally phenotypic mutant born from a collection of inbred mice that exhibit ageing alterations, such as age-dependent early onset of senile amyloidosis, degenerative arthropathy, cataracts, osteoporosis and osteoarthritis, reduced fecundity, early loss of fecundity and a shorter life span (death typically occurs at 8 months) [24]. The SAMR1 mice come from a collection of three long-lived control strains [24]. SAMR1 and SAMP8 mice were raised on an AKR/J genetic background.

**Fig. 3:**
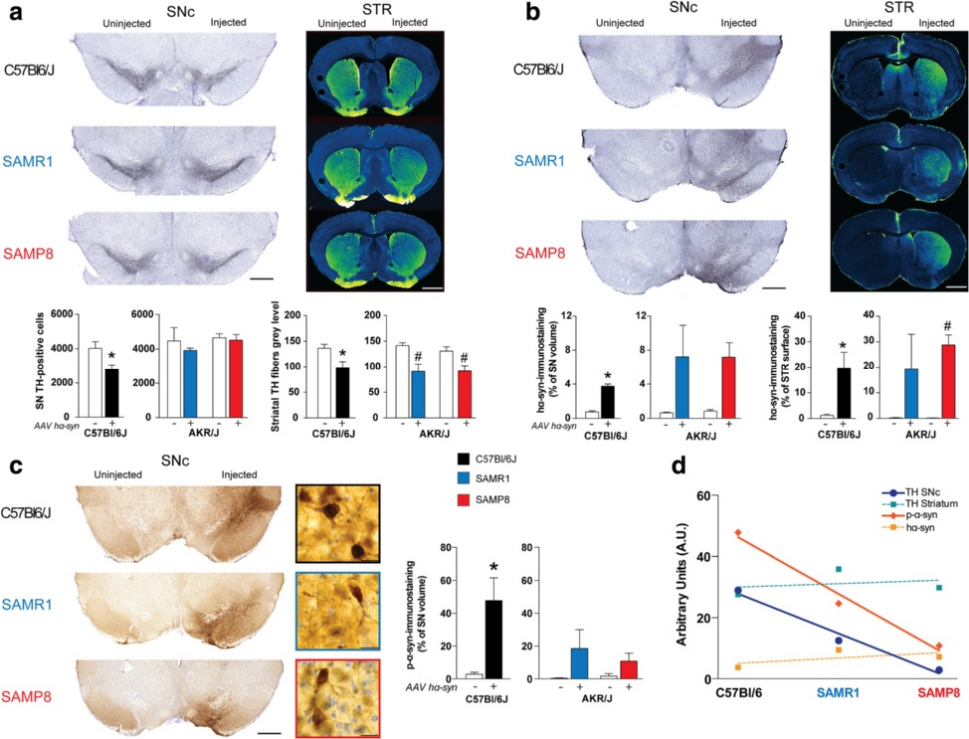
Human α-syn overexpression induces dopaminergic neurodegeneration associated with α-syn pathology in mice. **a** Stereological cell counts of SNpc TH-positive neurons (*left* two panels, *n* = 4 animals for each genotype) and mean grey values of striatal TH immunoreactivity (*right* two panels) at 20 weeks after surgery compared to non-injected side (white bars). Top panels display representative TH immunostaining at SNpc (*left*) and striatum (*right*) levels in the three mouse strains. **b** Volume quantification of the hα-syn immunostaining at the SNpc level (left two panels) and surface quantification of the hα-syn immunostaining at the striatal level (right two panels) at 20 weeks after surgery. Top panels display representative hα-syn immunostaining at SNpc (left) and striatum (*right*) levels in the mouse strains used. **c** Volume quantification of the p-α-syn immunostaining at the SNpc level at 20 weeks after surgery. Left panels display representative p-α-syn immunostaining at SNpc at macroscopic (*left*) and microscopic (*right*) levels in all mouse strains. **d** Covariance analysis of each parameter measured in all mouse strains. Data regarding TH pathway are expressed as a percentage of loss of TH fibers or cell bodies. Syn data are expressed as a percentage of immunopositive surface of the structure of interest. STR: striatum; * *p* < 0.05 vs non injected side of C57Bl/6 J mice; # *p* < 0.05 vs non injected side of the AKR/J background mice concerned. Scales: 1.5 mm for STR, 0.5 mm for SNpc and 20 μm for high magnification pictures of the C panel. hα-syn: human α-syn; p-α-syn: S129 phosphorylated α-syn

The integrity of the nigrostriatal pathway was assessed at 20 weeks after stereotactic surgery to cover the life span of SAMP8 mice. While all strains displayed a significant decrease in striatal TH immunoreactivity, only C57Bl/6 J mice presented a significant decrease in the number of TH-positive neurons in the SNpc (Fig. 3a). SAMP8 and SAMR1 mice, on the AKR/J background, are less susceptible to hα-syn-induced degeneration than C57Bl/6 J mice. Whether this difference actually represents a delayed degeneration or an absence of degeneration remains to be fully established.

We then examined the occurrence and pattern of hα-syn pathology at both striatal and nigral levels. All strains demonstrated strong hα-syn staining at both levels (Fig. 3b). In contrast to rats, p-α-syn staining was not restricted to SNpc (Fig. 3c). Further examination revealed an intriguing pattern for p-α-syn staining: C57Bl/6 J exhibited the strongest pattern and SAMP8 the weakest (Fig. 3c). Interestingly, p-α-syn levels and the number of TH-positive cells in the SNpc were covariant: the strain with the highest amount of p-α-syn (i.e., C57Bl/6 J) was also the one that displayed the highest dopamine neuron death (Fig. 3d).

To establish that SAMP8 and SAMR1 mice were less susceptible to PD-related neurodegeneration, animals were treated with a subacute regimen of intoxication with 1-methyl-1,2,3,6-tetrahydropyridine (MPTP), a pro-parkinsonian mitochondrial neurotoxin that reproduces several aspects of PD [25]. Only C57Bl/6 J mice displayed significant DA neurodegeneration 5 months following MPTP intoxication, a result reminiscent of our observation in AAV-hα-syn injected animals (Additional file 11: Figure S7). This result supports our hypothesis of a lack of sensitivity of SAMP8 and SAMR1 mice, mostly attributable to their AKR/J genetic background.

Taken together, our results in mice are three-fold. First, we established that overexpression of hα-syn induces neurodegeneration associated with accumulation of α-syn and p-α-syn in mice. Second, phenotypic mutants with either accelerated senescence or resistance to senescence did not display differential susceptibility to hα-syn overexpression and MPTP intoxication. Third, in mice, p-α-syn levels correlated with nigrostriatal degeneration.

### Ageing does not enhance susceptibility to α-syn overexpression in marmoset monkeys

Non-human primate neuronal physiology is closer to human than rodents. We thus took advantage of aged and young adult marmoset monkeys to decipher whether ageing might increase the susceptibility of dopamine neurons to degenerate following AAV-hα-syn injection.

**Fig. 4:**
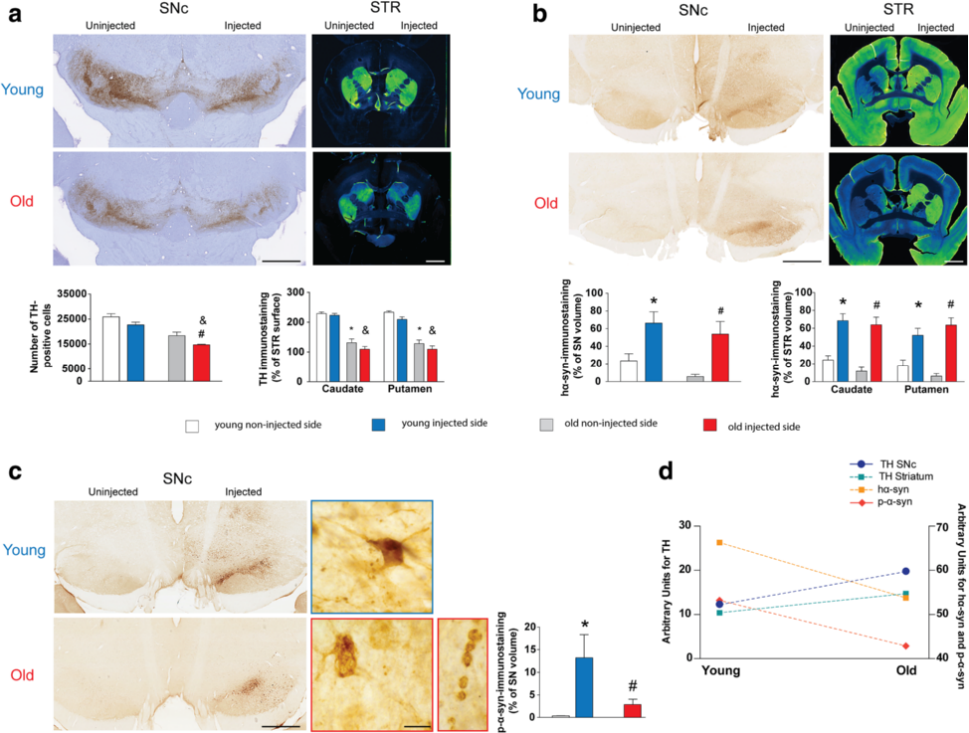
Human α-syn overexpression induces dopaminergic neurodegeneration associated with α-syn pathology in young and old marmoset monkeys. **a** Stereological cell counts of SNpc TH-positive neurons (left panel, *n* = 8 for young animals and *n* = 5 for old animals) and mean grey values of striatal TH-positive fibers (right panel) at 11 weeks after surgery. Colors: blue: injected side of young animals; red: injected side of old animals; white and light grey: non-injected side of young and old animals, respectively. Top panels display representative TH immunostaining at SNpc (*left*) and striatum (*right*) levels. **b** Volume quantification of the hα-syn immunostaining at the SNpc level (left panel) and surface quantification of the hα-syn immunostaining at the striatal level (*right* panel) at 11 weeks after surgery. Top panels display representative hα-syn immunostaining at SNpc (*left*) and striatum (*right*) levels. **c** Volume quantification of the p-α-syn immunostaining at the SNpc level at 11 weeks after surgery. Left panels display representative human p-α-syn immunostaining at SNpc at macroscopic (*left*) and microscopic (*right*) levels. **d** Covariance analysis of each parameter measured in young and old marmosets. Data regarding TH pathway represent a loss of TH fibers or cell bodies. STR: striatum; * *p* < 0.05 vs non injected side of young animals; # *p* < 0.05 vs non injected side of old animals; & *p* < 0.05 vs injected side of young animals. Scales: 1.5 mm for STR, 0.5 mm for SNpc, 250 μm and 20 μm for high magnification pictures of the (**c**) panel. hα-syn: human α-syn; p-α-syn: S129 phosphorylated α-syn

The integrity of the nigrostriatal pathway was investigated in young adult (1–2 years old, *n* = 8) and old marmosets (>6 years old, *n* = 5). The life span is around 10 years, i.e., the old animals had exceeded their mid-life expectancy. In humans, PD typically occurs around 60 years of age. Sham-operated old animals displayed a significant reduction of TH immunoreactivity in the striatum (40 % in the caudate nucleus and 43 % in the putamen) associated with a significant decrease of TH-positive cells in the SNpc (29 %) compared to sham-operated young animals (Fig. 4a). Animals were euthanized at 11 weeks post-surgery to avoid any losses due to death from natural causes of some old animals. AAV-hα-syn induced a degeneration of TH-positive neurons of the same magnitude (13 % in young animals, 20 % in old animals, *p* = 0.7182). At the striatal level, hα-syn overexpression produced depletion in striatal TH-positive fibers in both caudate nucleus (8 % for young and 11 % for old animals – Fig. 4a) and putamen (23 % for young animals and 15 % for old animals – Fig. 4a). These results indicate that hα-syn-induced degeneration of the nigrostriatal pathway was not affected by the age of the animals.

We then investigated the occurrence and pattern of hα-syn pathology. The level of overexpression and pattern of hα-syn staining were identical at both SNpc and striatal levels in both groups (around 300 % - Fig. 4b). Then, we examined p-α-syn immunostaining in the SNpc (Fig. 4c). While both young and old animals showed p-α-syn immunoreactivity only on the injected side, old animals exhibited a significantly lower staining than young animals (Fig. 4c). In contrast to mice (Fig. 3c) but in agreement with our results in rat (Additional file 8: Figure S4), p-α-syn staining was mostly restricted to SNpc and could be found in neuronal perikarya and dendritic terminals (Fig. 4c). Nevertheless, p-α-syn and the number of TH-positive neurons in the SNpc (Fig. 4d) did not covary, unlike in mice (Fig. 3d). Both age groups presented an identical level of nigrostriatal degeneration regardless of the p-α-syn staining levels. This last observation raises questions as to the interpretation of p-α-syn levels in different species.

## Discussion

Here we report that AAV2/9-mediated p.A53T mutated human α-syn overexpression induces robust nigrostriatal neurodegeneration in mice, rats and monkeys, establishing translational comparisons among species. In addition, the comprehensive degeneration kinetics and high-resolution kinematic characterizations in the rat model uncovered clinically relevant relationships between the extent of the lesion and the behavioural PD-like phenotype.

Previous studies have shown that overexpression of human WT α-syn in rats induces around 80 % of dopamine neuron loss after 16 weeks [19], a result comparable to the present p.A53T mutant α-syn overexpression in the rat (80 % of DA cell loss after 16 weeks) and paralleled by only 30 % dopamine neuron loss in mice after 20 weeks and 20 % in aged marmoset monkeys after 11 weeks. Our results in the rat model are in agreement with previous reports [10, 13] using the same expression cassette, although the level and time frame of neurodegeneration varies depending on the titer and vector serotype used [10]. Patients harbouring p.A53T mutation do not present a classic parkinsonism but an early-onset, fast-progressing disease associated with secondary symptoms [26]. Of interest, the dynamics of WT α-syn overexpression [19] fit with our degeneration curve (Fig. 1f) although one study reports a faster degeneration time course for p.A53T mutant α-syn overexpression [20]. In mice, one study did not show any significant differences between WT and p.A53T mutated α-syn overexpression [9]. Such differences might be attributable to the use of different expression cassettes, as it appears in other reports that dose of vector could be more important than the α-syn mutation [9, 22]. The present data suggest that this mutation might not have the same functional consequences in rodents and marmoset monkeys as in humans. The reason for the discrepancy between different clinical symptoms and identical rates of degeneration in preclinical models remains to be established. The presence of an alanine residue at position 53 is not fully conserved during evolution. Several species, including rodents and marmoset monkeys, exhibit a threonine instead of an alanine residue at position 53 in their endogenous α-syn [27, 28]. Investigation in a mammalian model that does not have the A53T substitution (such as macaques) might resolve this apparent discrepancy.

The behavioural phenotype of AAV-hα-syn-injected rats shares many features with the human disease. In the open field and locomotor tasks, AAV-hα-syn-injected rats displayed both akinesia and bradykinesia. L-3,4-dihydroxyphenylalanine treatment effectively alleviates these symptoms [15]. Here, we quantified these deficits using objective and quantitative analyses. We found that a decrease in velocity (gait hypokinesia) and abnormal gait, as evidenced by impaired inter-limb coordination and increased double stance were the main features altered in response to α-syn over-expression. These features constitute the hallmarks of shuffling gait in PD patients [26]. The longitudinal follow-up of basic motor function allowed us to establish that motor dysfunction appears from 4 weeks onwards, when 60 % of dopamine neurons and 80 % of striatal dopaminergic fibers have been lost. These results are in perfect coherence with the known or hypothesized relationship between nigrostriatal lesions and symptoms in MPTP-treated macaques [29] and PD humans, respectively [30]. Applying the regression defined for the AAV-hα-syn-injected rats to the AAV-hα-syn-injected mice and monkeys, for which we have a single time point, led us to predict that symptoms would be detected, post-surgery, at 133 weeks in mice, 244 weeks for young marmosets and 142 weeks for old marmosets. While such duration would exceed the mouse life span, rendering the model inadequate for behavioural studies, the durations are indeed compatible with marmoset life span. These extrapolations suggest that age may impact upon the development of symptoms, which remain to be confirmed. However, this long-term window excludes the involvement of marmoset monkeys in behavioural studies, restricting the use of this species for studies relying on post-mortem endpoints. Previous work suggested that, after the first occurrence of clinical signs, the evolution of the disease slows down [30]. Contrary to the MPTP-treated monkey model, in which degeneration of dopamine neurons fits with a linear regression [29, 30], AAV-hα-syn injection in the rat followed an exponential regression equation characteristic of a fast onset of neurodegeneration followed by a slow evolution, a feature to bear in mind both for the study of the prodromal phase and for the validation of therapeutic approaches.

Several studies have reported the existence of p-α-syn in brains from PD patients [21]. α-syn could indeed be phosphorylated at S129 in preclinical models of PD [19, 31, 32]. However, it remains unclear from in vitro and in vivo data whether phosphorylation of α-syn impacts upon its fibrillation process [33]. We here show that mice exhibiting the highest p-α-syn levels display the highest level of degeneration. However, the marmoset monkey data contradict this hypothesis as no correlation between the p-α-syn levels and DA degeneration was found in this species. Several other reports have already questioned the relevance of p-α-syn to the neurodegenerative process and as a therapeutic target [34]. Our data suggest that p-α-syn might have different impact depending upon the species, calling for caution in interpreting data obtained in rodents compared to primates. It may be noted that p-α-syn is found in healthy aged human brains [35].

A key objective of the present study was to address the role of ageing in the susceptibility of dopamine neurons to degeneration in mice and marmoset monkeys. In mice, aged animals are more prone to MPTP-induced striatal dopamine loss [36]. The absence of neurodegeneration after hα-syn overexpression or MPTP administration observed in SAMP8 and SAMR1 mice might be due to their AKR/J background, which is known to be less sensitive to MPTP than C57Bl/6 J [22]. Whether this is indeed a lack of sensitivity or the consequence of an unknown neuroprotective factor remains to be established. Similar to the differential rat strain susceptibility, these results call for caution regarding genetic background when using genetically engineered or mutant mice for studying PD pathogenesis. A previous study using MPTP in marmoset monkeys reported that aged marmosets (8–10 years-old) were no more susceptible to MPTP intoxication than younger animals [37], a finding consistent with our results using α-syn-induced neurodegeneration. In rhesus monkeys, aged animals (20–23 years-old) required a third as much MPTP to induce the same behavioural phenotype as in young animals [38], suggesting an age-dependent susceptibility. The differential MPTP susceptibility between these two species might be due to the presence of neuromelanin, a pigment that accumulates over time in the SNpc through oxidation of dopamine to aminochrome and polymerization, in macaques but not in marmosets [39]. Melanized neurons are more susceptible than non-melanized neurons both in PD patients [40] and MPTP-treated primates [41]. Further studies are needed to unravel whether the presence of neuromelanin in aged macaques, and to a lesser extent in young macaques, might impact the degeneration course following hα-syn overexpression. The specificity of primate brain for the accumulation of neuromelanin strengthens the special relevance of preclinical studies in primates for PD. Nevertheless, ageing here appears as a modest independent factor that only decreases the gap to a putative threshold for the appearance of PD symptoms in marmoset monkey. Our results thus strengthen the multiple-hit hypothesis [4, 42]. We propose that a combination of deleterious events accelerate the ageing-related decline in dopamine neuron function, rather than the stochastic acceleration hypothesis in which ageing directly sensitizes the dopamine system [3].

## Conclusions

Ageing might only be a contributing factor to the extent of hα-syn-induced degeneration, suggesting that mature adult animals might be enough for testing disease-modifying therapies. Since PD patients would only benefit from such therapy after symptom onset, i.e., when they already have a loss of 70-80 % of striatal dopaminergic terminals and 50-60 % of dopamine neurons, the present study defines the most suitable time points for therapeutic intervention in those three models. Considering the identified dynamics (Fig. 1), we propose that starting administration from 3–4 weeks after surgery in the AAV-hα-syn rat model would be most clinically-relevant, offering translatable behavioural and post-mortem endpoints while the mouse and marmoset models should be used for post-mortem endpoints only.

### Availability of supporting data

The data sets supporting the results of this article are included within the article and its additional files.

## Additional files.

Additional file 1: Table S1. Computed kinematic and kinematic variables. Additional file 2: Figure S6. Extraction of gait parameters affected by hα-syn overexpression in rats. PC analysis was applied on all gait parameters measured during overground locomotion, as reported in Fig. 2. Parameters correlating with PC1 (|loading factor| > 0.5) and thus specifically altered by hα-syn overexpression were regrouped into functional clusters, which we named for clarity. The numbers refer to Table S1. Some of the parameters with high factor loadings are reported in the histogram plots of Fig. 2. Additional file 3: Table S2. List of antibodies used in the study Additional file 4: Table S3. Sampling and size of probes used in the study. Additional file 5: Figure S1. rAAV2/9 vector-mediated overexpression of hα-syn in rat SNpc induces progressive dopaminergic neurodegeneration related to hα-syn expression dynamics. (a-e) Histogram plots represent mean values of DA related (a-c) or hα-syn (d,e) related markers. *: *p* < 0.05 vs sham animals, $ : *p* < 0.05 vs 72 h, # : *p* < 0.05 vs 1 week. Errors bars are SEM. hα-syn: human α-syn. Additional file 6: Figure S2. Striatal and SNpc hα-syn expression. Delay between SNpc (a) and striatal (b) hα-syn expression. Hα-syn pattern in the SNpc starting from a strong cellular staining (a – early time points) to a more diffuse one (a – late timepoints). In the striatum, morphological change of hα-syn staining from a long-wire staining (b - early timepoints) to a more punctiform one (b – late time points). Scale bars: (a) 50 μm, (b) 20 μm. hα-syn: human α-syn. Additional file 7: Figure S3. Presence of ubiquitin-hα-syn colocalization in SNpc remaining neurons. (a,e,i) TH staining, (b,f,j) ubiquitin, (c,g,k) hα-syn (d,h,l) merge. (a-h) ×10 magnification, (i-l) ×40 magnification. hα-syn: human α-syn Additional file 8: Figure S4. p-α-syn staining in the SNpc. (a-d) Representative photomicrographs of p-α-syn staining in the SNpc of sham (a) or injected (b-d) animals. In contrast to hα-syn staining, p-α-syn is highly localized in neuronal perikarya (c) and neurites (d) at 16 weeks after surgery. (e) Quantification of the SNpc volume positive for p-α-syn staining. Scale bar: (a,b) 1 mm, (c) 20 μm. hα-syn: human α-syn, pα-syn: S129 phosphorylated α-syn. Additional file 9: Figure S5. Wistar rats are significantly less sensitive to hα-syn overexpression than Sprague Dawley rats. Histogram plots represent number of TH-positive cells in the SNpc at 8 weeks after surgery for sham (white bars) and AAV-hα-syn injected rats (black bars). ***: *p* < 0.01 vs control side, #: *p* < 0.05. Error bars: SEM. Additional file 10:
**Movie 1.** Locomotion across an horizontal ladder in sham and AAV-hα-syn-injected rats. This movie shows a representative kinematic recording of quadrupedal locomotion along the equally spaced rungs of an horizontal ladder in a sham operated and an AAV-hα-syn-injected rat. Additional file 11: Figure S7. SAMP8 and SAMR1 mice do not present nigrostriatal degeneration after MPTP intoxication while C57Bl/6 J does. Histogram plots represent the number of remaining TH-positive cells in the SNpc 21 days after the last injection of MPTP. *: *p* < 0.05 vs non injected animals.

## Abbreviations

α-syn: Alpha-synuclein
AAV: Recombinant adeno-associated virus
DA: Dopamine
DAT: Dopamine transporter
hα-syn: Human mutant p.A53T alpha-synuclein
LB: Lewy bodies
LN: Lewy neurites
MPTP: 1-methyl-1,2,3,6-tetrahydropyridine
PC: Principal component
PD: Parkinson’s disease
SAMP8: Senescence-accelerated mouse-prone 8
SAMR1: Senescence-accelerated mouse-resistant 1
SNpc: Substantia nigra pars compacta
TH: Tyrosine Hydroxylase
WRPE: Woodchuck hepatitis virus post-transcriptional regulatory element
WT: Wild-type
