# Supplementary material for: Lack of additive role of ageing in nigrostriatal neurodegeneration triggered by α-synuclein overexpression
Source: Acta Neuropathol Commun. 2015 Jul 25;3:46. doi: 10.1186/s40478-015-0222-2 (PMC4513748; doi:10.1186/s40478-015-0222-2)
Supplement: Additional file 1: Table S1. — Computed kinematic and kinematic variables. [file 40478_2015_222_MOESM1_ESM.docx]

|  |  |  |  |  |  |
| --- | --- | --- | --- | --- | --- |
|  | **#** | **DETAILED EXPLANATION OF VARIABLE** |  |  |  |
|  |  |  |  |  |  |
|  | **TEMPORAL FEATURES OF GAIT** | |  |  |  |
|  | **1** | Cycle duration (s) |  |  |  |
|  | **2** | Cycle velcity (cm/s) |  |  |  |
|  | **3** | Stance duration (s) |  |  |  |
|  | **4** | Swing duration (s) |  |  |  |
|  | **5** | Relative stance duration (% of cycle duration) |  |  |  |
|  |  |  |  |  |  |
|  | **LIMB ENDPOINT TRAJECTORIES** | |  |  |  |
|  | **6** | Stride length (cm) |  |  |  |
|  | **7** | Step length (cm) |  |  |  |
|  | **8** | 3D limb endpoint path length (cm) |  |  |  |
|  | **9** | Maximal backward position with respect to pelvis (cm) |  |  |  |
|  | **10** | Minimum forward position with respect to pelvis (cm) |  |  |  |
|  | **11** | Step height (cm) |  |  |  |
|  | **12** | Maximal speed during swing (cm/s) |  |  |  |
|  | **13** | Relative timing of max velocity during swing |  |  |  |
|  | **14** | Acceleration at swing onset (cm/s^2) |  |  |  |
|  | **15** | Endpoint velocity (cm/s) |  |  |  |
|  | **16** | Orientation of the velocity vector at swing onset (deg) |  |  |  |
|  |  |  |  |  |  |
|  | **STABILITY** | | |  |  |
|  | **17** | Double stance duration (s) | Base of support |  |  |
|  | **18** | Posititioning of the foot at stance onset with respect to pelvis (cm) |  |  |  |
|  | **19** | Stance width (cm) |  |  |  |
|  | **20** | Pelvis maximum lateral movement (deg) |  |  |  |
|  | **21** | Pelvis minimum lateral movement (deg) |  |  |  |
|  | **22** | pelvis lateral movement amplitude (deg) |  |  |  |
|  |  |  |  |  |  |
|  | **23** | Variability of saggital trunk oscillation | Trunk and pelvic position and oscillations |  |  |
|  | **24** | Variability in velocity of saggital trunk oscillation |  |  |  |
|  | **25** | Varibility of vertical mid-point hip oscillation |  |  |  |
|  | **26** | Variability of medio-lateral mid-point hip oscillation |  |  |  |
|  | **27** | Variability of medio-lateral hip rotations |  |  |  |
|  |  |  |  |  |  |
|  | **28** | Forward motion of body center of mass (cm) | Displacement of body mid-point |  |  |
|  | **29** | Medio-lateral motion of body center of mass (cm) |  |  |  |
|  | **30** | Vertical motion of body center of mass (cm) |  |  |  |
|  | **31** | 3D motion of body center of mass (cm) |  |  |  |
|  |  |  |  |  |  |
|  | **JOINT ANGLES AND SEGMENTAL OSCILLATIONS** | | |  |  |
|  | **32** | Crest oscillations (deg) | forward oscillation of limb segments |  |  |
|  | **33** | Thigh oscillations (deg) |  |  |  |
|  | **34** | Shank oscillations (deg) |  |  |  |
|  | **35** | Foot oscillations (deg) |  |  |  |
|  | **36** | Whole limb oscillations (deg) |  |  |  |
|  |  |  |  |  |  |
|  | **37** | Crest oscillations (deg) | backward oscillation of limb segments |  |  |
|  | **38** | Thigh oscillations (deg) |  |  |  |
|  | **39** | Shank oscillations (deg) |  |  |  |
|  | **40** | Foot oscillations (deg) |  |  |  |
|  | **41** | Whole limb oscillations (deg) |  |  |  |
|  |  |  |  |  |  |
|  | **42** | Hip joint (deg) | Extension of joint angles |  |  |
|  | **43** | Knee joint (deg) |  |  |  |
|  | **44** | Ankle joint (deg) |  |  |  |
|  |  |  |  |  |  |
|  | **45** | Whole limb abduction (deg) | Abduction |  |  |
|  | **46** | Foot abduction (deg) |  |  |  |
|  |  |  |  |  |  |
|  | **47** | Hip joint (deg) | Flexion of joint angles |  |  |
|  | **48** | Knee joint (deg) |  |  |  |
|  | **49** | Ankle joint (deg) |  |  |  |
|  |  |  |  |  |  |
|  | **50** | Whole limb abduction (deg) | Adduction |  |  |
|  | **51** | Foot adduction (deg) |  |  |  |
|  |  |  |  |  |  |
|  | **52** | Crest oscillations (deg) | Amplitude of segmental and joint oscillations (Max-min) |  |  |
|  | **53** | Thigh oscillations (deg) |  |  |  |
|  | **54** | Shank oscillations (deg) |  |  |  |
|  | **55** | Foot oscillations (deg) |  |  |  |
|  | **56** | Whole limb oscillations (deg) |  |  |  |
|  |  |  |  |  |  |
|  | **57** | Hip joint (deg) |  |  |  |
|  | **58** | Knee joint (deg) |  |  |  |
|  | **59** | Ankle joint (deg) |  |  |  |
|  |  |  |  |  |  |
|  | **60** | Whole limb medio-lateral oscillation (deg) |  |  |  |
|  | **61** | Foot abduction/adduction (deg) |  |  |  |
|  |  |  |  |  |  |
|  | **VELOCITY** | | |  |  |
|  | **62** | Whole limb oscillation velocity (deg/s) | Minimum angle velocity (deg/s) |  |  |
|  | **63** | Hip joint angle oscillation velocity (deg/s) |  |  |  |
|  | **64** | Knee joint angle oscillation velocity (deg/s) |  |  |  |
|  | **65** | Ankle joint angle oscillatino velocity (deg/s) |  |  |  |
|  |  |  |  |  |  |
|  | **66** | Whole limb oscillation velocity (deg/s) | Maximum angle velocity (deg/s) |  |  |
|  | **67** | Hip joint angle oscillation velocity (deg/s) |  |  |  |
|  | **68** | Knee joint angle oscillation velocity (deg/s) |  |  |  |
|  | **69** | Ankle joint angle oscillatino velocity (deg/s) |  |  |  |
|  |  |  |  |  |  |
|  | **70** | Whole limb oscillation velocity (deg/s) | Amplitude of angle velocity (Max - Min) deg/s |  |  |
|  | **71** | Hip joint angle oscillation velocity (deg/s) |  |  |  |
|  | **72** | Knee joint angle oscillation velocity (deg/s) |  |  |  |
|  | **73** | Ankle joint angle oscillatino velocity (deg/s) |  |  |  |
|  |  |  |  |  |  |
|  | **COORDINATION** | | |  |  |
|  | **74** | Degree of linear coupling between hindlimb elevation angles | PC analysis |  |  |
|  |  |  |  |  |  |
|  | **75** | Temporal coupling between crest and thigh oscillation | FFT decomposition |  |  |
|  | **76** | Temporal coupling between thigh and leg oscillation |  |  |  |
|  | **77** | Temporal couling between leg and foot oscillation |  |  |  |
|  |  |  |  |  |  |
|  | **78** | Correlation between crest and thigh oscillation | Cross-correlation between segments |  |  |
|  | **79** | Correlation between thigh and leg oscillation |  |  |  |
|  | **80** | Correlation between leg and foot oscillation |  |  |  |
|  | **81** | Correlation between hip and knee oscillation |  |  |  |
|  | **82** | Correlation between knee and ankle oscillation |  |  |  |
|  |  |  |  |  |  |
|  | **83** | Temporal lag between backward positions of crest and thigh oscillations | Relative coupling between segments |  |  |
|  | **84** | Temporal lag between forward positions of crest and thigh oscillations |  |  |  |
|  | **85** | Temporal lag between backward positions of thigh and leg oscillations |  |  |  |
|  | **86** | Temporal lag between forward positions of the thigh and leg oscillations |  |  |  |
|  | **87** | Temporal lag between backward positions of leg and foot oscillations |  |  |  |
|  | **88** | Temporal lag between forward positions of leg and foot oscillations |  |  |  |
|  |  |  |  |  |  |
|  | **89** | Correlation between whole hindlimb oscillations | Left-right |  |  |
|  |  |  |  |  |  |
|  |  |  |  |  |  |
|  |  |  |  |  |  |
|  |  |  |  |  |  |
|  |  |  |  |  |  |
