# Supplementary figures and images for: Lack of additive role of ageing in nigrostriatal neurodegeneration triggered by α-synuclein overexpression
Source: Acta Neuropathol Commun. 2015 Jul 25;3:46. doi: 10.1186/s40478-015-0222-2 (PMC4513748; doi:10.1186/s40478-015-0222-2)

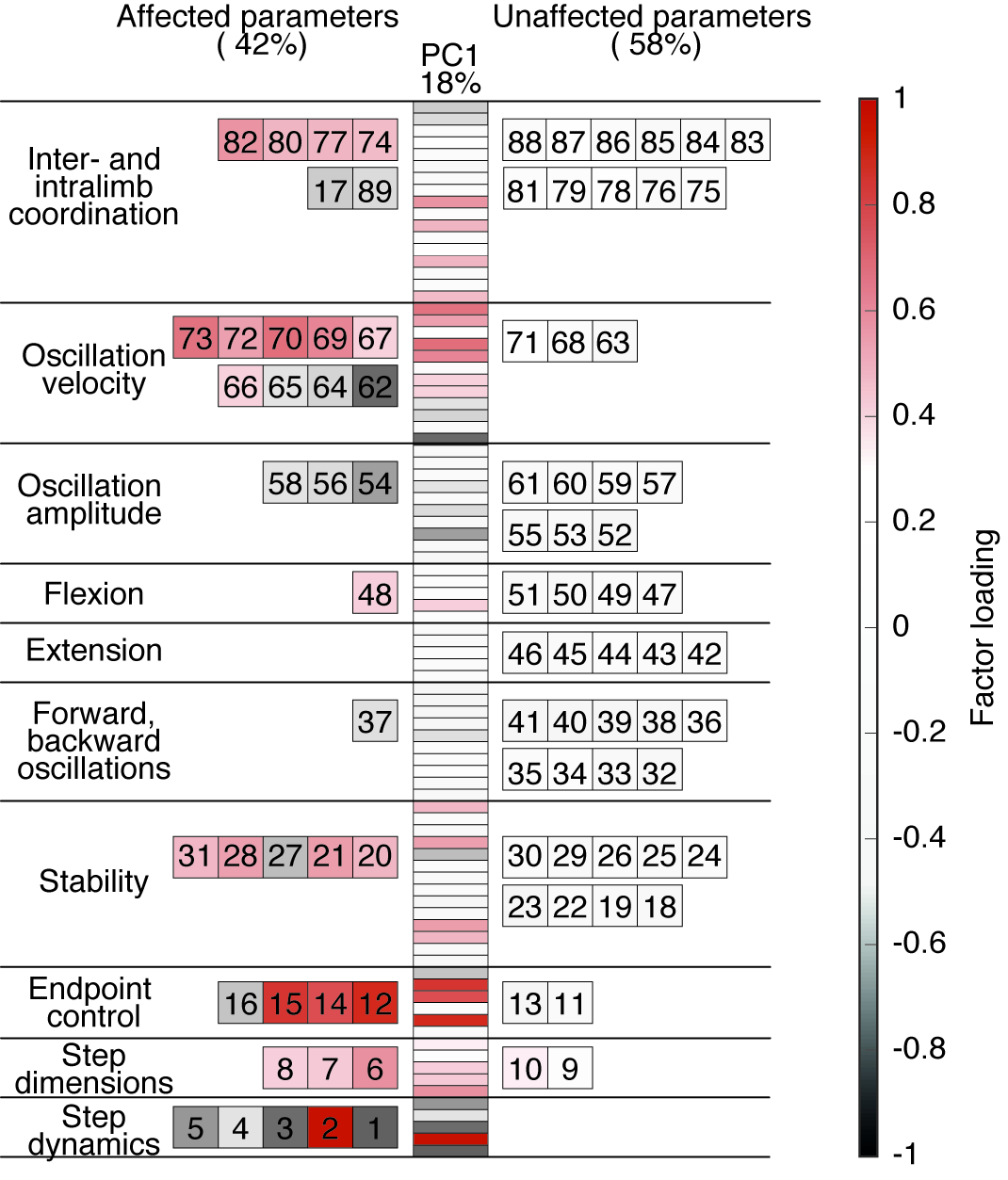

Supplement: Additional file 2: Figure S6. — Extraction of gait parameters affected by hα-syn overexpression in rats. PC analysis was applied on all gait parameters measured during overground locomotion, as reported in Fig. 2. Parameters correlating with PC1 (|loading factor| > 0.5) and thus specifically altered by hα-syn overexpression were regrouped into functional clusters, which we named for clarity. The numbers refer to Table S1. Some of the parameters with high factor loadings are reported in the histogram plots of Fig. 2. [file 40478_2015_222_MOESM2_ESM.tif]

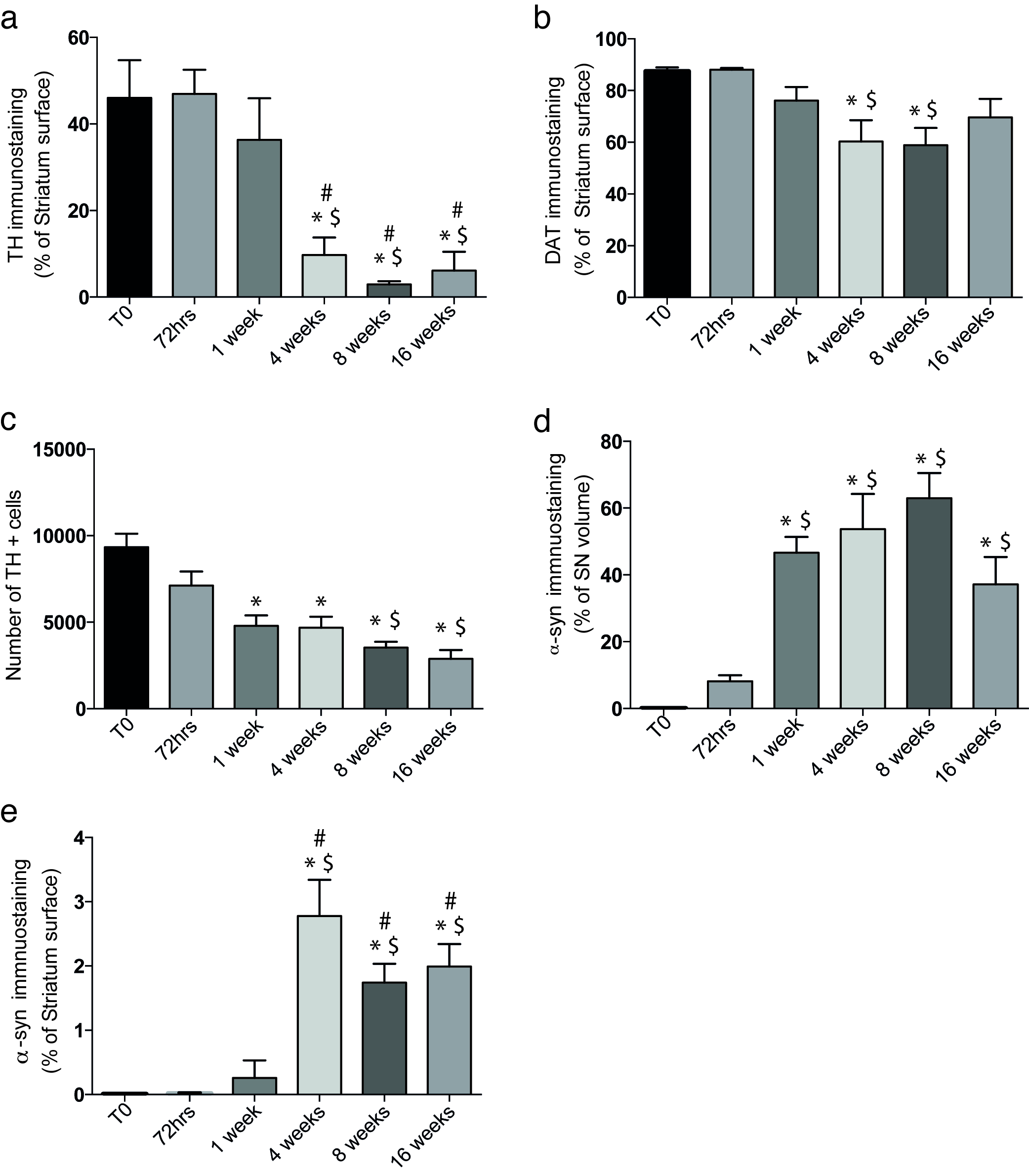

Supplement: Additional file 5: Figure S1. — rAAV2/9 vector-mediated overexpression of hα-syn in rat SNpc induces progressive dopaminergic neurodegeneration related to hα-syn expression dynamics. (a-e) Histogram plots represent mean values of DA related (a-c) or hα-syn (d,e) related markers. *: p < 0.05 vs sham animals, $ : p < 0.05 vs 72 h, # : p < 0.05 vs 1 week. Errors bars are SEM. hα-syn: human α-syn. [file 40478_2015_222_MOESM5_ESM.tif]

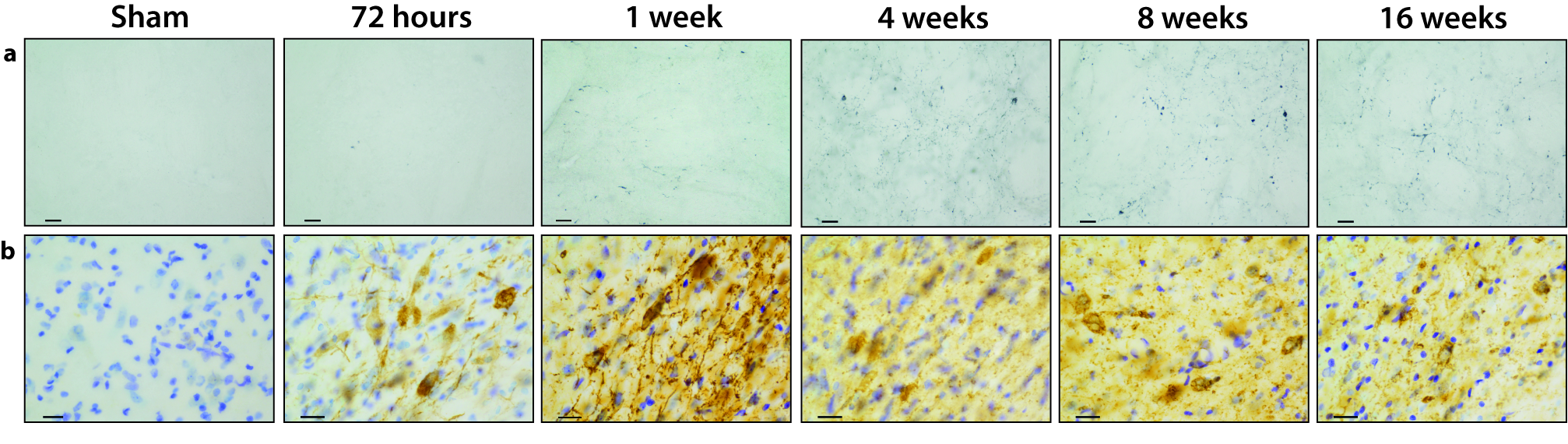

Supplement: Additional file 6: Figure S2. — Striatal and SNpc hα-syn expression. Delay between SNpc (a) and striatal (b) hα-syn expression. Hα-syn pattern in the SNpc starting from a strong cellular staining (a – early time points) to a more diffuse one (a – late timepoints). In the striatum, morphological change of hα-syn staining from a long-wire staining (b - early timepoints) to a more punctiform one (b – late time points). Scale bars: (a) 50 μm, (b) 20 μm. hα-syn: human α-syn. [file 40478_2015_222_MOESM6_ESM.tif]

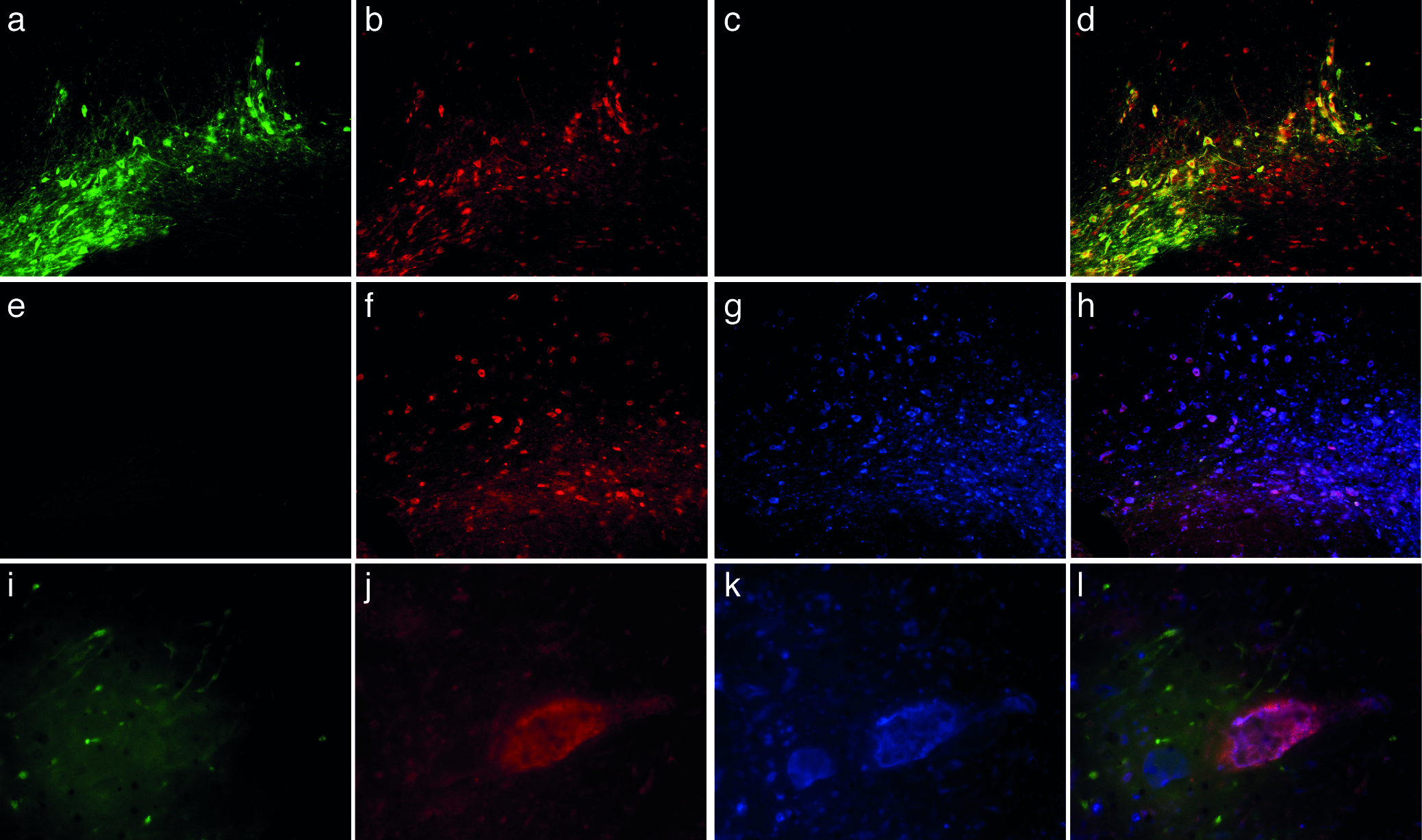

Supplement: Additional file 7: Figure S3. — Presence of ubiquitin-hα-syn colocalization in SNpc remaining neurons. (a,e,i) TH staining, (b,f,j) ubiquitin, (c,g,k) hα-syn (d,h,l) merge. (a-h) ×10 magnification, (i-l) ×40 magnification. hα-syn: human α-syn [file 40478_2015_222_MOESM7_ESM.tif]

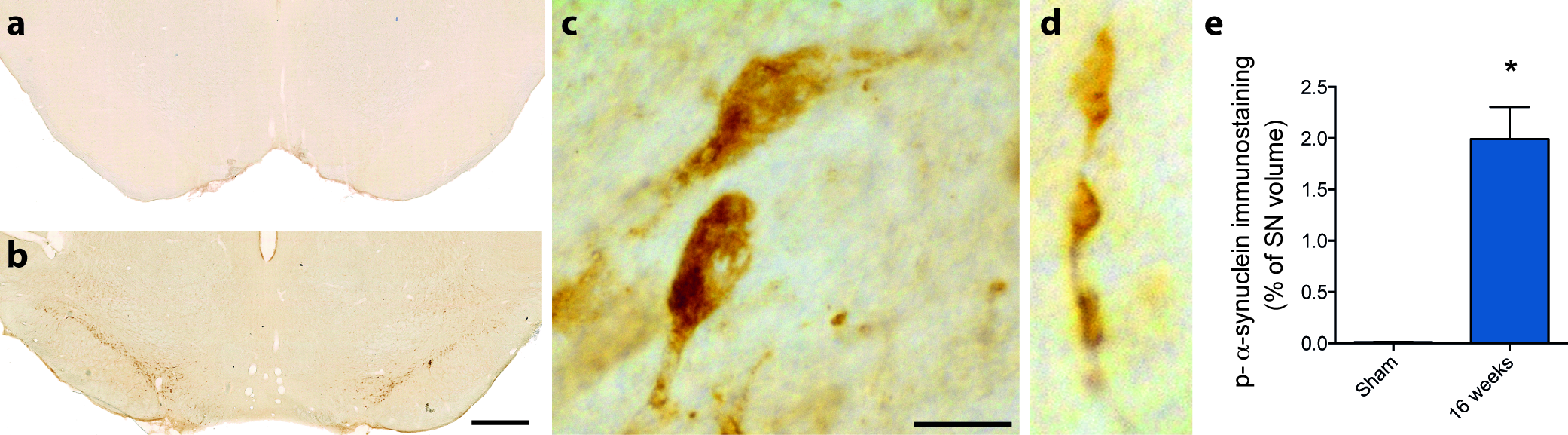

Supplement: Additional file 8: Figure S4. — p-α-syn staining in the SNpc. (a-d) Representative photomicrographs of p-α-syn staining in the SNpc of sham (a) or injected (b-d) animals. In contrast to hα-syn staining, p-α-syn is highly localized in neuronal perikarya (c) and neurites (d) at 16 weeks after surgery. (e) Quantification of the SNpc volume positive for p-α-syn staining. Scale bar: (a,b) 1 mm, (c) 20 μm. hα-syn: human α-syn, pα-syn: S129 phosphorylated α-syn. [file 40478_2015_222_MOESM8_ESM.tif]

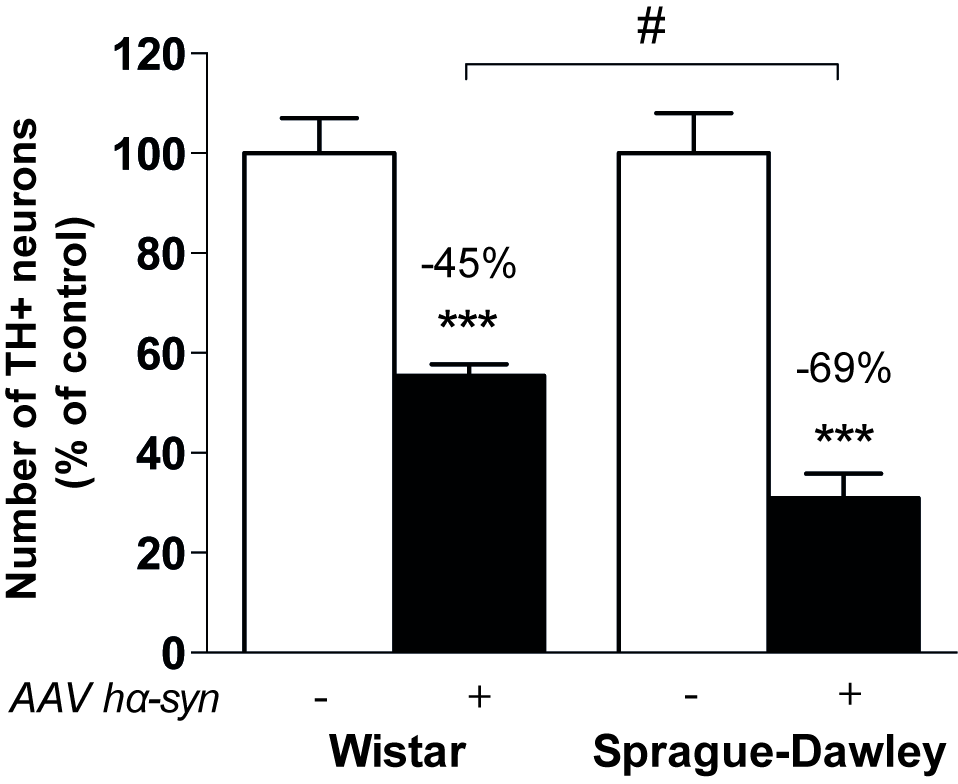

Supplement: Additional file 9: Figure S5. — Wistar rats are significantly less sensitive to hα-syn overexpression than Sprague Dawley rats. Histogram plots represent number of TH-positive cells in the SNpc at 8 weeks after surgery for sham (white bars) and AAV-hα-syn injected rats (black bars). ***: p < 0.01 vs control side, #: p < 0.05. Error bars: SEM. [file 40478_2015_222_MOESM9_ESM.tif]

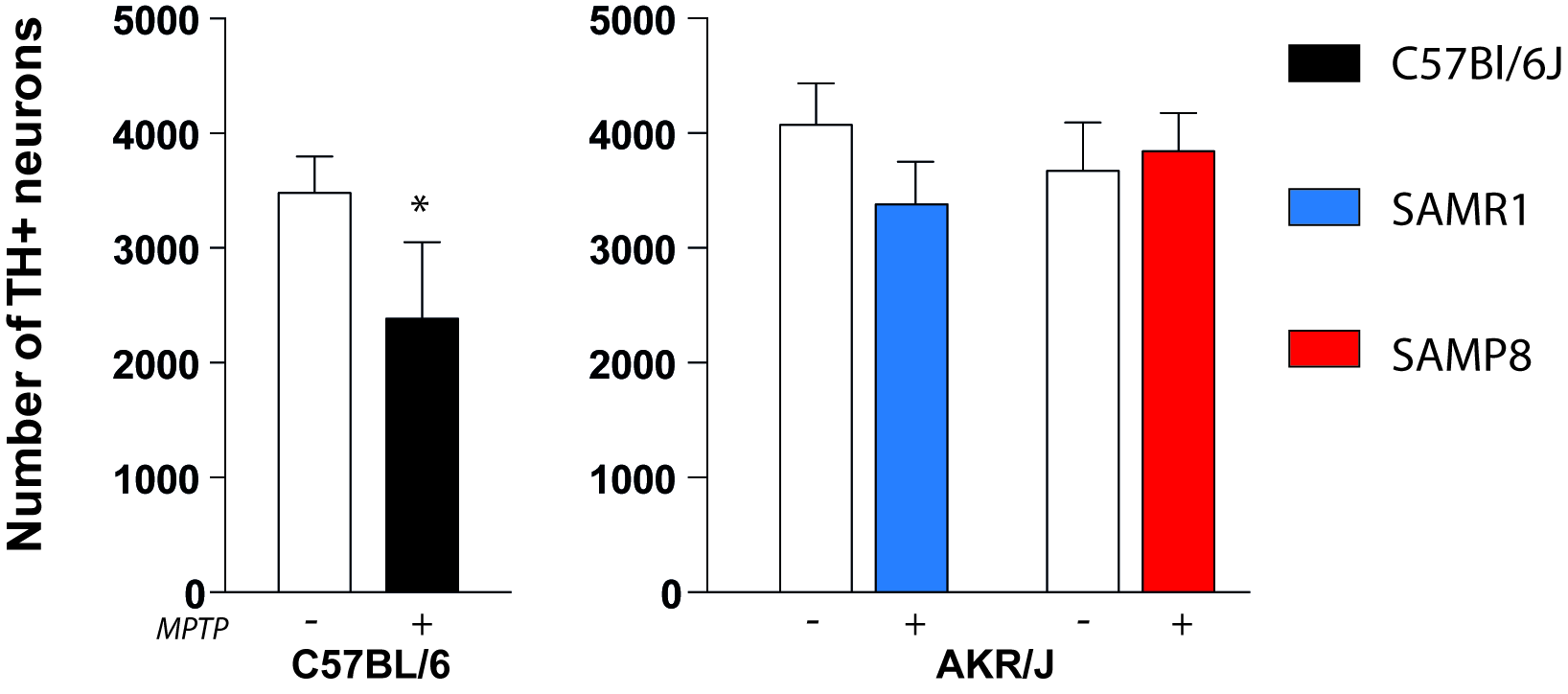

Supplement: Additional file 11: Figure S7. — SAMP8 and SAMR1 mice do not present nigrostriatal degeneration after MPTP intoxication while C57Bl/6 J does. Histogram plots represent the number of remaining TH-positive cells in the SNpc 21 days after the last injection of MPTP. *: p < 0.05 vs non injected animals. [file 40478_2015_222_MOESM11_ESM.tif]
