# Supplementary material for: Lack of additive role of ageing in nigrostriatal neurodegeneration triggered by α-synuclein overexpression
Source: Acta Neuropathol Commun. 2015 Jul 25;3:46. doi: 10.1186/s40478-015-0222-2 (PMC4513748; doi:10.1186/s40478-015-0222-2)
Supplement: Additional file 3: Table S2. — List of antibodies used in the study [file 40478_2015_222_MOESM3_ESM.docx]

| **Antibody** | **Company** | **Reference** | **Host** | **Dilution** |
| --- | --- | --- | --- | --- |
| Tyrosine Hydroxylase (TH) | Millipore | MAB318 | Mouse | 1/5000 3 no |
| Tyrosine Hydroxylase (TH) | Abcam | AB113 | Sheep | 1/1000 1no |
| Transporter Dopamine (DAT) | Home-made | Ref. 18 | Rabbit | 1/5000 3 no |
| Human α-syn | Thermo Scientific | MS1572 | Mouse | 1/1000 1no |
| α-syn phosphorylated at Ser129 | Abcam | AB51253 | Rabbit | 1/500 3 no |
| Ubiquitin | Sigma | U5379 | Rabbit | 1/1000 1 no |
