# Supplementary material for: Lack of additive role of ageing in nigrostriatal neurodegeneration triggered by α-synuclein overexpression
Source: Acta Neuropathol Commun. 2015 Jul 25;3:46. doi: 10.1186/s40478-015-0222-2 (PMC4513748; doi:10.1186/s40478-015-0222-2)
Supplement: Additional file 4: Table S3. — Sampling and size of probes used in the study. [file 40478_2015_222_MOESM4_ESM.docx]

| **Species** | **SN Sampling** | **Probe (µm)** | **Space (µm)** |
| --- | --- | --- | --- |
| Mouse | 1/4 | 50x40 | 150x120 |
| Rat | 1/6 | 80x60 | 240x180 |
| Marmoset | 1/6 | 80x60 | 240x180 |
